# Routine Culture–Resistant *Mycobacterium tuberculosis* Rescue and Shell-Vial Assay, France

**DOI:** 10.3201/eid2511.190431

**Published:** 2019-11

**Authors:** Mustapha Fellag, Jamal Saad, Nicholas Armstrong, Eric Chabrière, Carole Eldin, Jean-Christophe Lagier, Michel Drancourt

**Affiliations:** Institut Hospitalier Universitaire Méditerranée Infection, Marseille, France (M. Fellag, J. Saad);; Aix-Marseille University, Marseille (M. Fellag, J. Saad, N. Armstrong, E. Chabrière, C. Eldin, J.-C. Lagier, M. Drancourt)

**Keywords:** *Mycobacterium tuberculosis*, culture, shell-vial assay, tuberculosis, tuberculosis and other mycobacteria, antimicrobial resistance, France, bacteria

## Abstract

We used shell-vial assay with a medium that buffered rifampin to isolate routine culture–resistant *Mycobacterium tuberculosis* bacteria from cerebrospinal fluid and rifampin-containing intervertebral disc and vertebral corpus of a patient in treatment for Pott’s disease and disseminated tuberculosis. Whole-genome sequencing confirmed *M. tuberculosis* lineage 4 (Euro-American) strain.

Culturing *Mycobacterium tuberculosis* from clinical specimens confirms the viability of mycobacteria and enables drug susceptibility testing (*1*). Routinely used culture protocols may fail to isolate *M. tuberculosis* from vertebral biopsy specimens in 17%–50% of cases (*2*) and from cerebrospinal fluid (CSF) specimens in >80% of cases (*3*). However, the shell-vial culture assay (*4*) has demonstrated a high sensitivity for the isolation of mycobacteria, representing an alternative method for growing *M. tuberculosis* (*5**,**6*).

We report a case of disseminated tuberculosis (TB) documented by culturing *M. tuberculosis* strain P7739 from a patient who was previously treated with antituberculous drugs. We used the shell-vial assay, even though strain P7739 resisted standard cell-free culture techniques. The Ethics Committee of Institut Hospitalier Universitaire Méditerranée Infection (Marseille, France) approved this study (no. 2016-024, October 19, 2016).

A 47-year-old man who was HIV negative and had no previous history of TB received a diagnosis of Pott’s disease with systemic tuberculosis on the basis of clinical symptoms of spondylodiscitis, myelitis, meningitis, and pulmonary miliary infection. In January 2017, the patient suffered lumbar pain; a computer tomodensitometry scan showed corporeal bone defects in the L1 vertebra. Ten months later, magnetic resonance imaging revealed T12–L1 vertebral spondylodiscitis with a paravertebral abscess in the right iliopsoas muscle. We performed 2 biopsies of the vertebral corpus of T12 and L1 and 1 biopsy of the intervertebral disc; we also collected CSF and sputum. These clinical specimens remained negative for *M. tuberculosis* using microscopic examination after Ziehl-Neelsen staining, real-time PCR (GenExpert, https://www.cepheid.com), culture in liquid medium BBL Mycobacteria Growth Indicator Tube (Becton Dickinson, https://www.bd.com), and in solid culture media including Coletsos medium (bioMérieux, https://www.biomerieux.com). Thirteen days later, the neurologic condition of the patient deteriorated with meningeal syndrome. Examination by magnetic resonance imaging showed a triventricular hydrocephalus and transependymal periventricular resorption, which led to an emergency external ventricular bypass. We strongly suspected TB, so we introduced TB treatment (900 mg rifampin, 300 mg isoniazid, 1,800 mg pyrazinamide, and 1,200 mg ethambutol daily). We performed a second round of clinical sampling from bronchoalveolar fluid, T12 vertebral corpus and T12–L1 intervertebral discs, and CSF 9 days after the initiation of TB treatment. Microscopic examination after Ziehl-Neelsen staining remained negative, as did results from the GenExpert assay, except for the detection of a rifampin-susceptible *M. tuberculosis* complex mycobacterium in 3 vertebral biopsy specimens and the intervertebral disc specimen. Culturing in MGIT tubes and on Coletsos remained negative after 8 weeks of incubation. 

We inoculated 5 samples (1 CSF, 3 bone biopsy, 1 intervertebral disc) on human embryonic lung (HEL) fibroblasts (HEL 299 ATCC CCL-137; American Type Culture Collection, https://www.lgcstandards-atcc.org) using the shell-vial assay incorporating negative controls, as described previously (*6*). After 17–28 days of incubation, negative control vials remained sterile, whereas these 5 inoculated cell cultures grew Ziehl-Neelsen–positive mycobacteria (Figure). Seven-day subculture on Coletsos medium yielded colonies identified as *M. tuberculosis* strain P7739 by matrix-assisted laser desorption/ionization time-of-flight mass spectrometry (*7*)*.* In vitro susceptibility assays indicated susceptibility to rifampin, ethambutol, chloramphenicol, clofazimine, and trimethoprim/sulfamethoxazole and resistance to minocycline and pyrazinamide (*8*). The genome sequence of *M. tuberculosis* strain P7739 (1 scaffold with 4,392,478 bp and 64.8% guanine-cytosine content) mapped more closely with *M. tuberculosis* Erdman (GenBank accession no. NC_020559.1) using CLC Genomics version 7 (https://www.qiagenbioinformatics.com). Annotation using Prokka version 1.12 https://github.com/tseemann/prokka) yielded 4,171 protein-coding genes, 2 repeat regions, and 55 RNA genes (51 tRNA, 3 rRNA, and 1 tmRNA). Single-nucleotide polymorphism (SNP) comparison with different lineages at the core genome level using TB profiler (http://tbdr.lshtm.ac.uk) indicated that strain P7739 (GenBank accession no. CAABOY010000000) was related to *M. tuberculosis* lineage 4 and did not encode for antimicrobial resistance–associated mutations.

**Figure:**
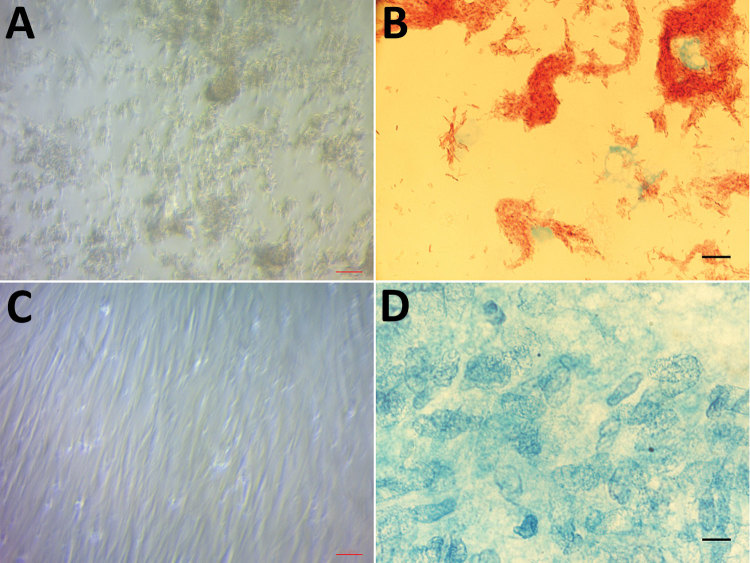
Light microscopic examination of human embryonic lung cells inoculated for 28 days from a clinical sample from a 47-year-old man with Pott’s disease and systemic tuberculosis, France. A) Cytopathic effect consisting of cell lysis caused by growing *Mycobaterium tuberculosis.* Original magnification ×200. B) *M. tuberculosis* mycobacteria observed after Ziehl-Neelsen staining. Original magnification ×1,000, by oil immersion. C) Absence of any cytopathic effect in negative control cell culture. Original magnification ×200. D) Absence of any mycobacteria in the negative control cell culture after Ziehl-Neelsen staining. Original magnification ×1,000 by oil immersion.

We isolated *M. tuberculosis* strain P7739 from this patient using the shell-vial assay 9 days after initiation of TB treatment. Liquid chromatography mass spectrometry (LC/MS) (Acquity I-Class Vion-IMS Q-Tof, Waters, https://www.waters.com) detected 0.45 µg rifampin/g in the intervertebral disc specimen and 0.04 µg rifampin/g in the 2 vertebral bone biopsy specimens; 1 exhibited anti-TB activity. Further dosage with rifampin at 0, 7, and 14 days postincubation with HEL revealed that 80% of the amount of free rifampin was lost at day 14 (Appendix Figure).

Gouriet et al. reported that 11.5% of *Mycobacterium* sp. isolates are cultured only in a cell culture assay (*6*). We cultured *M. tuberculosis* using the shell-vial assay after incubating for 17–28 days, in accordance with previous studies (*5*). Our findings confirm the use of the shell-vial assay in diagnosis of tuberculosis in patients in whom TB is suspected and whose specimens do not grow on conventional media, especially after initiating TB treatment in patients before sampling. Our observations suggest that cell culture medium buffers the anti-TB activity of clinical specimens, thus enabling growth of *M. tuberculosis* mycobacteria that are no longer exposed to anti-TB activity.

Appendix Additional information about routine culture–resistant *Mycobacterium tuberculosis* rescue using shell-vial assay.
