# Supplementary material for: Routine Culture–Resistant Mycobacterium tuberculosis Rescue and Shell-Vial Assay, France
Source: Emerg Infect Dis. 2019 Nov;25(11):2131–3. doi: 10.3201/eid2511.190431 (PMC6810202; doi:10.3201/eid2511.190431)
Supplement: Appendix — Additional information about routine culture–resistant Mycobacterium tuberculosis rescue using shell-vial assay. [file 19-0431-Techapp-s1.pdf]

Article DOI: https://doi.org/10.3201/eid2511.190431

Routine Culture–Resistant *Mycobacterium tuberculosis* Rescue and Shell-Vial Assay, France

Appendix

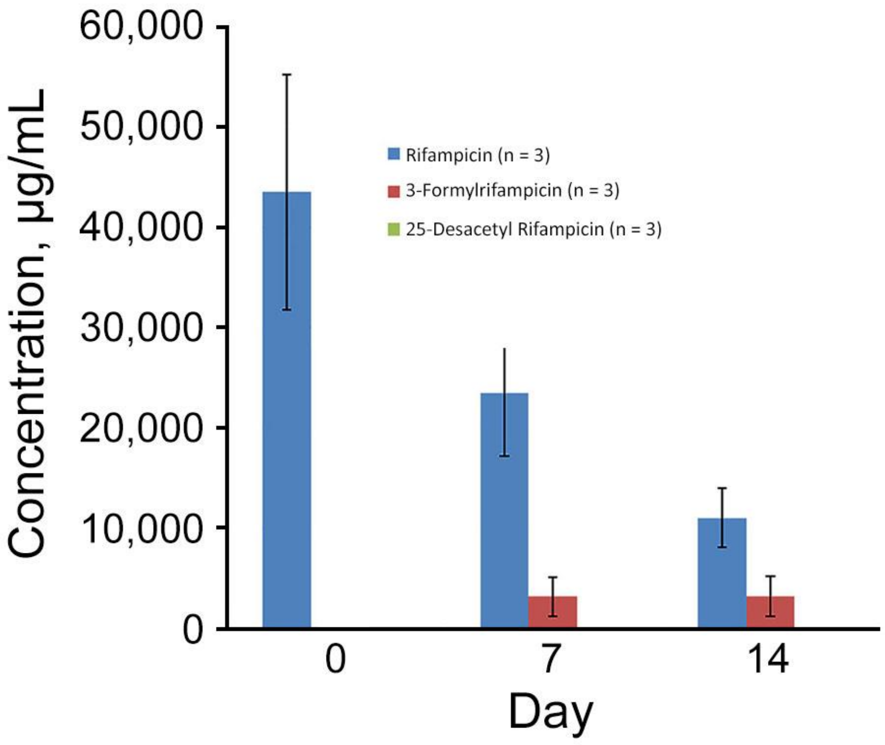

Appendix Figure. Ion response of free rifampin and its human metabolites measured over 14 days by liquid chromatography mass spectrometry in the supernatant of HEL cells. Bars indicate the standard deviation (n = 3) for each metabolite.
